# Altered expression of MX2 and SAMD4A in PBMCs predicts early treatment responses in HBeAg-positive chronic hepatitis B patients during Peg-IFN-α therapy

**DOI:** 10.3389/fphar.2026.1844257

**Published:** 2026-06-22

**Authors:** Hao Pang, Qiqi Zhang, Lüping Chen, Bo Qin

**Affiliations:** 1 Department of Infectious Diseases, Chongqing Key Laboratory of Infectious Diseases and Parasitic Diseases, The First Affiliated Hospital of Chongqing Medical University, Chongqing, China; 2 Central Laboratory, The First Affiliated Hospital of Chongqing Medical University, Chongqing, China

**Keywords:** hepatitis B virus, MX2, Peg-IFN-α, response, Samd4a

## Abstract

**Background:**

Chronic Hepatitis B (CHB) is a major global health concern. This study aimed to evaluate whether myxovirus resistance 2 (MX2) and sterile alpha motif domain containing 4A (SAMD4A) mRNA levels in peripheral blood mononuclear cells (PBMCs) can predict early treatment responses to pegylated interferon-alpha (Peg-IFN-α) in hepatitis B e antigen (HBeAg)-positive CHB patients.

**Methods:**

This prospective cohort study enrolled HBeAg-positive CHB patients who received Peg-IFN-α for 48 weeks. Patients were classified into virological response (VR; HBV DNA <50 IU/mL at week 48) and non-virological response (NVR) groups, and into serological response (SR; HBeAg seroconversion at week 48) and non-serological response (NSR) groups. MX2 and SAMD4A mRNA levels in PBMCs were measured by quantitative real-time PCR (qRT-PCR) at weeks 0, 12, and 24. Spearman’s rank correlation analysis was performed to evaluate the relationships between gene expression levels and declines in HBeAg and HBV DNA. Univariate and multivariate logistic regression analyses were conducted to identify independent predictors of VR and SR, and predictive performance was assessed using receiver operating characteristic (ROC) curves with calculation of the area under the curve (AUC).

**Results:**

At week 48, the VR and SR rates were 64.63% and 39.02%, respectively. Dynamic changes in MX2 and SAMD4A mRNA levels differed significantly between the VR and NVR groups and between the SR and NSR groups. Both MX2 and SAMD4A mRNA levels at weeks 12 and 24 were positively correlated with concurrent reductions in HBeAg and HBV DNA. Notably, early upregulation of both genes at week 12 was significantly associated with subsequent reductions in HBeAg and HBV DNA observed at week 24. Multivariate analysis identified MX2 and SAMD4A as independent predictors of both VR and SR at weeks 12 and 24. At week 24, the AUC values of MX2 were 0.7567 for VR and 0.8421 for SR, while those of SAMD4A were 0.8549 for VR and 0.8717 for SR.

**Conclusion:**

MX2 and SAMD4A are important biomarkers for early treatment responses to Peg-IFN-α in HBeAg-positive CHB patients.

## Introduction

Chronic Hepatitis B (CHB) continues to impose a heavy global burden, as persistent hepatitis B virus (HBV) infection drives progression to cirrhosis and hepatocellular carcinoma (HCC) in many individuals (Vittal et al., 2022; Yuen et al., 2018). The associated morbidity and mortality place considerable strain on healthcare systems worldwide (Danpanichkul et al., 2025).

The management of CHB relies primarily on pegylated interferon-alpha (Peg-IFN-α) and nucleos(t)ide analogs (NAs) (Jeng et al., 2023). While NAs effectively suppress viral replication, they rarely achieve functional cure, whereas Peg-IFN-α offers a finite treatment course with a higher likelihood of inducing sustained off-treatment response in selected patients (Song et al., 2021; He et al., 2022). Among hepatitis B e antigen (HBeAg)-positive CHB patients, those with persistently elevated alanine aminotransferase (ALT) levels typically exhibit high HBV DNA levels and are at increased risk of disease progression, thereby warranting antiviral intervention (Terrault et al., 2018; EASL, 2017; Huang et al., 2022; Huang et al., 2023). The occurrence of HBeAg seroconversion, defined as HBeAg loss and seroconversion to anti-HBe, is a critical clinical endpoint for HBeAg-positive CHB patients, as it reflects partial restoration of host immune control and is associated with a more favorable clinical course, including a reduced risk of cirrhosis and HCC, and a higher likelihood of subsequent HBsAg clearance (Moucari et al., 2009a; EASL, 2017). However, the rates of HBeAg seroconversion following Peg-IFN-α therapy remain limited, generally ranging from 30% to 40% (EASL, 2017; Terrault et al., 2016; Omata et al., 2017). Given the considerable cost, potential adverse effects, and heterogeneous response to interferon-based therapy, there is a compelling clinical need to identify reliable predictors of treatment response. Although several conventional predictors, including baseline hepatitis B surface antigen (HBsAg), HBV DNA, ALT, and HBV genotype, have demonstrated clinical utility in guiding interferon therapy, their predictive performance remains inconsistent across different patient populations and may not fully reflect host antiviral immune responses (Buster et al., 2009; Ye and Chen, 2021; Lebossé et al., 2017; Kramvis et al., 2022). Consequently, identifying additional biomarkers with improved predictive accuracy and stronger biological relevance is essential for predicting HBeAg seroconversion in Peg-IFN-α–treated patients with HBeAg-positive CHB.

Peg-IFN-α exerts its biological effects by specifically binding to the type I interferon receptor, composed of IFNAR1 and IFNAR2. This interaction activates the Janus kinase (JAK)/signal transducer and activator of transcription (STAT) pathway (Ivashkiv and Donlin, 2014; Liu et al., 2012), subsequently inducing the expression of over 300 interferon-stimulated genes (ISGs) (Sadler and Williams, 2008). Besides the immunomodulatory roles of specific ISGs expressed in immune cells, some ISGs exhibit direct antiviral activity within virally infected hepatocytes (Sadler and Williams, 2008). Studies have reported that specific ISGs inhibit HBV replication at different stages through both transcriptional and post-transcriptional mechanisms (Uprichard et al., 2003; Zhao et al., 2024). For instance, APOBEC3A mediates the deamination and degradation of covalently closed circular DNA (cccDNA) (Lucifora et al., 2014); STAT1 and PML interact with cccDNA minichromosomes to establish a suppressive epigenetic environment (Cheng et al., 2020); and TRIM5γ inhibits HBV transcription by targeting HBx for degradation (Tan et al., 2019). Additionally, recent research indicates that the expression levels of several ISGs in hepatic tissue or peripheral blood are closely associated with the therapeutic response to Peg-IFN-α in CHB patients (Shan et al., 2025; Han et al., 2017). Despite these advancements, the absence of reliable biomarkers for predicting treatment efficacy remains a significant challenge, and the specific roles of many ISGs in Peg-IFN-α–mediated viral control are still incompletely understood. Among potentially predictive ISGs, myxovirus resistance 2 (MX2) and sterile alpha motif domain containing 4A (SAMD4A) have emerged as promising candidates due to their distinct and complementary antiviral mechanisms and clinical relevance in liver disease.

MX2 is a dynamin-like guanosine triphosphatase whose expression is strongly upregulated by type I interferons (Goujon et al., 2013). Evidence suggests that MX2 possesses potent anti-HBV activity by diminishing HBV RNA levels and indirectly disrupting the formation of cccDNA (Wang et al., 2020). SAMD4A is located on chromosome 14q22.2 and encodes a 718-amino acid protein that shares homology with *Drosophila* Smaug1 (Smibert et al., 1996). The sterile alpha motif (SAM) domain within SAMD4A is a highly conserved structural feature that facilitates direct binding to RNA stem-loop structures, also known as Smaug recognition element (SRE) motifs (Baez and Boccaccio, 2005). In human subjects, SAMD4A serves as an important anti-HBV ISG that exhibits significant anti-HBV efficacy through its interaction with the conserved SRE found in HBV transcripts, subsequently leading to their degradation (Wang et al., 2021). Despite the important roles of these ISGs in modulating the HBV life cycle, their clinical utility as predictive biomarkers remains largely unexplored. Most previous studies investigating predictive biomarkers of interferon efficacy in CHB have primarily focused on classical ISGs, including MX1, STAT1, and SOCS3 (Han et al., 2017; Li et al., 2022). Although the predictive value of these conventional ISGs has been extensively investigated in the context of interferon therapy for CHB, their predictive performance has often varied across different patient cohorts, suggesting that additional host-response biomarkers with stronger biological relevance and improved clinical applicability may still be needed. In contrast, MX2 and SAMD4A regulate HBV replication through distinct antiviral mechanisms and may therefore reflect complementary aspects of interferon-induced host antiviral immunity. Notably, emerging evidence also suggests potential clinical relevance of these genes in chronic viral liver disease. MX2 expression has been shown to be upregulated in the livers of IFN-α–treated chronic hepatitis C patients and to correlate with biochemical indices and viral subtypes in hepatitis C virus (HCV) patients (Hevezi et al., 2011; He et al., 2023). Furthermore, the expression level of SAMD4A correlates with viral load and HBV sensitivity in HBV-infected patients (Wang et al., 2021). Importantly, despite their potential biological and clinical relevance, no study has systematically investigated the dynamic changes in MX2 and SAMD4A mRNA levels in peripheral blood mononuclear cells (PBMCs) during Peg-IFN-α therapy, nor their associations with treatment outcomes and their predictive value for interferon efficacy in HBeAg-positive CHB patients. Systematic investigation of these dynamic changes is particularly important because the host immune response to Peg-IFN-α is time-dependent (Micco et al., 2013; Reijnders et al., 2011). Capturing real-time fluctuations in ISG expression provides a more accurate reflection of the evolving antiviral state than static baseline measurements, which is crucial for early prediction of treatment efficacy and facilitating response-guided therapy. In addition, given that PBMC-based biomarkers offer a minimally invasive and readily accessible alternative to liver tissue sampling, elucidating their clinical relevance is of considerable translational importance.

Therefore, this study aims to determine the dynamic expression of these ISGs and their associations with treatment efficacy and HBeAg seroconversion in patients diagnosed with HBeAg-positive CHB undergoing interferon-based antiviral regimens. By elucidating this relationship, we aim to identify potential novel biomarkers that can predict the chance of a patient having an HBeAg seroconversion, ultimately contributing to more precise and individualized therapeutic strategies.

## Methods

### Patient recruitment

In this prospective cohort study, a total of 82 HBeAg-positive CHB patients (aged 18–65 years) who had received at least 48 weeks of Peg-IFN-α therapy were recruited between October 2023 and October 2024 at the Department of Infectious Diseases of the First Affiliated Hospital of Chongqing Medical University. A flowchart illustrating the study design is provided in Supplementary Figure S1. The diagnosis and management of these patients were conducted in accordance with the 2022 guidelines issued by the Chinese Society of Hepatology and the Chinese Society of Infectious Diseases (You et al., 2023). The inclusion criteria were as follows: (1) HBsAg positivity for at least 6 months; (2) HBeAg positivity with concurrent anti-HBe negativity; (3) serum HBV DNA levels >1 × 10^5^ IU/mL; (4) ALT levels ≥2 and <10 × the upper limit of normal (ULN); and (5) no prior history of antiviral therapy. Patients were excluded based on the following criteria: (1) coinfection with other hepatotropic viruses, including HCV or hepatitis D virus (HDV); (2) coinfection with other clinically relevant viral pathogens, such as human immunodeficiency virus (HIV) or Epstein–Barr virus (EBV); (3) receipt of immunosuppressive therapy within the preceding 6 months; (4) presence of other inflammatory or autoimmune disorders, including rheumatoid arthritis or autoimmune hepatitis; (5) evidence of advanced liver injury or alternative liver diseases, such as alcoholic liver disease or metabolic dysfunction-associated fatty liver disease (MAFLD); (6) significant alcohol consumption (>20 g/day for women or >30 g/day for men); (7) decompensated cirrhosis or a history of malignancy; (8) severe psychiatric disorders, including uncontrolled epilepsy or major depressive disorder; (9) pregnancy or breastfeeding; and (10) any contraindications to interferon-based therapy. The study protocol was approved by the Ethics Committee of the First Affiliated Hospital of Chongqing Medical University (Reference number: 2023-311). In addition to the Peg-IFN-α–treated cohort, 46 untreated CHB patients and 34 healthy controls were recruited for baseline comparisons.

Serum HBsAg levels were measured at baseline and every 12 weeks during Peg-IFN-α therapy. Peripheral blood samples were obtained at weeks 0, 12, and 24. These time points were selected in view of the established role of weeks 12 and 24 as clinically relevant early on-treatment milestones for evaluating interferon response and viral kinetics during Peg-IFN-α therapy, whereas baseline sampling was used to assess pretreatment gene expression status (EASL, 2017; You et al., 2023). PBMCs were isolated, and the intracellular mRNA expression levels of MX2 and SAMD4A were determined. Patients were categorized according to treatment outcomes as follows: (1) Virological response (VR) group: defined as an undetectable HBV DNA level (<50 IU/mL) at week 48; patients failing to meet this criterion were classified as the non-virological response (NVR) group; (2) Serological response (SR) group: defined as HBeAg seroconversion (characterized by HBeAg loss accompanied by the development of anti-HBe antibodies) at week 48; patients who did not meet this criterion were classified as the non-serological response (NSR) group.

### Clinical and laboratory measurements

Serum HBV DNA levels were quantified using a fluorescence real-time PCR assay with a lower detection limit of 50 IU/mL (Cobas Z480 system; Roche, Switzerland). Serological markers, including HBsAg, hepatitis B surface antibody (anti-HBs), HBeAg, and hepatitis B e antibody (anti-HBe), were measured using the Abbott Architect i2000 immunoassay system. Biochemical parameters reflecting liver function were determined with an automated clinical chemistry analyzer (Cobas, Roche, Shanghai), while routine hematological indices were obtained using an automated blood cell analyzer (Mindray BC-6600, Shanghai).

### RNA extraction and quantitative real-time PCR (qRT-PCR)

Total RNA was isolated from PBMCs using TRIzol reagent and reverse-transcribed into complementary DNA (cDNA) using a commercial synthesis kit. The mRNA expression levels of MX2 and SAMD4A were subsequently quantified using a two-step qRT-PCR approach on a Bio-Rad CFX96 real-time PCR system (Bio-Rad, USA). Cycling conditions included an initial denaturation at 95 °C for 30 s, 40 cycles of 95 °C for 5 s and 60 °C for 30 s, followed by a melt curve analysis and a final cooling step (see Supplementary Methods for details). The expression levels of the target genes were assessed using relative quantification with GAPDH as the reference gene. Primer sequences used for qRT-PCR analysis are listed in Supplementary Figure S1. The relative mRNA expression levels of MX2 and SAMD4A were calculated using the comparative cycle threshold (2^−ΔΔCt^) method (Livak and Schmittgen, 2001).

### Statistical analysis

All statistical analyses and data visualization were performed using SPSS software (version 27.0) and GraphPad Prism (version 10.1.2). Prior to statistical testing, the serum concentrations of HBsAg, HBeAg, and HBV DNA were logarithmically transformed. Quantitative data are presented as mean ± standard deviation (SD) for normally distributed variables, and as median with interquartile range (IQR) for non-normally distributed variables. Longitudinal changes were analyzed using repeated-measures ANOVA (parametric) or the Friedman test (non-parametric). Between-group comparisons were performed using the Student’s t-test or the Mann–Whitney U test, as appropriate. Correlations were assessed using Pearson’s correlation coefficient for normally distributed data and Spearman’s rank correlation coefficient for non-normally distributed data. Univariate and multivariate logistic regression analyses were conducted to identify independent predictors associated with VR and SR. The predictive performance of MX2 and SAMD4A was assessed using receiver operating characteristic (ROC) curves with calculation of the area under the curve (AUC), and the DeLong test was used to compare differences between AUC values. To mitigate the risk of overfitting and assess the stability of the predictive models, internal validation was performed for the week 24 prediction models using bootstrap resampling with 1,000 iterations to estimate optimism-corrected model performance. Model discrimination was quantified using the concordance index (C-index), while calibration was evaluated using calibration curves, calibration intercepts, calibration slopes, and Brier scores. In addition, the potential clinical utility of the predictive models was assessed using decision curve analysis (DCA). A two-tailed P value <0.05 was considered statistically significant.

## Results

### Baseline characteristics and treatment responses

The baseline characteristics of each group are detailed in Table 1. Our study cohort comprised 34 healthy controls, 46 untreated CHB patients, and 82 HBeAg-positive CHB patients receiving Peg-IFN-α therapy. At baseline, no significant differences were observed between the untreated and treated CHB groups in age, gender, HBsAg, HBeAg, HBV DNA, ALT levels, WBC counts, or PLT counts. Similarly, age, gender, ALT levels, WBC counts, and PLT counts did not differ significantly across all three groups. Among the 82 HBeAg-positive CHB patients treated with Peg-IFN-α, virological and serological responses were assessed after 48 weeks of therapy. As shown in Supplementary Figure S2, 53 patients (64.63%) achieved the VR, and 32 patients (39.02%) achieved the SR.

**TABLE 1:** Baseline characteristics in the groups.

Characteristics	HC	Untreated CHB	CHB treated with peg-IFN-α	P Value
Number (n)	34	46	82	​
Age (year)	36.53 ± 11.99	42.63 ± 9.641	44.50 (37.75,55.00)	0.1083
Gender (male/female)	18/16	22/24	40/42	0.2891
HBsAg (log10 IU/mL)	UD	2.929 (2.506, 3.136)	3.034 (2.068, 3.415)	0.2874
HBV DNA (log10 IU/mL)	UD	5.669 (4.866, 6.601)	5.943 (5.215, 6.639)	0.1051
HBeAg (log10 IU/mL)	UD	2.427 (1.852, 2.776)	2.571 ± 0.7623	0.1822
ALT (U/L)	23.03 ± 7.022	146.7 ± 29.08^a^	138.5 (121.5, 161.0)^a^	0.3923
WBC (×10^9^/L)	6.025 ± 1.582	4.295 (3.665, 5.868)	4.050 (3.160, 5.615)	0.1075
PLT (×10^9^/L)	185.1 ± 41.53	178.0 (136.0, 196.3)	156.7 ± 65.19	0.1600

HBsAg: hepatitis B surface antigen; HBeAg: hepatitis B e antigen; ALT: alanine aminotransferase; WBC: white blood cells; PLT: platelet; HC: healthy controls; The results are presented as the median inter-quartile range or mean ± standard deviation; a Indicates a statistically significant difference compared with the HC, group (P < 0.05); UD: undetected.

### Reduced MX2 and SAMD4A mRNA levels in PBMCs are associated with HBV infection

**FIGURE 1:**
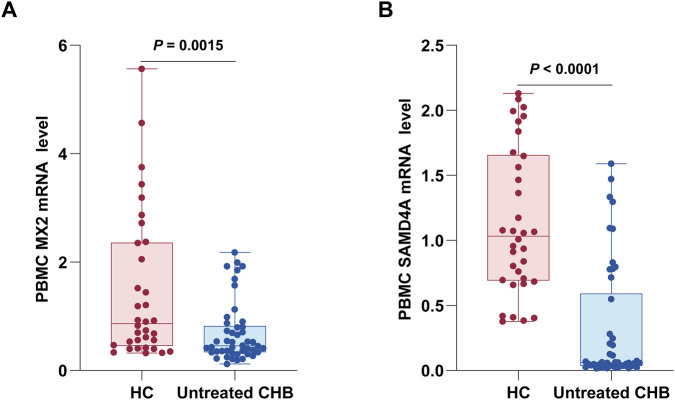
MX2 and SAMD4A mRNA expression in PBMCs of untreated CHB patients and healthy controls. MX2 **(A)** and SAMD4A **(B)** mRNA levels in PBMCs were analysed by qRT-PCR. The expression levels were calculated using the 2^-ΔΔCt^ method (Livak method), with GAPDH as the reference gene. P < 0.05 was statistically significant.

To investigate the impact of HBV on host ISG expression, we quantified MX2 and SAMD4A mRNA levels in PBMCs obtained from healthy controls and untreated CHB patients using qRT-PCR. Compared with healthy controls, the mRNA levels of both MX2 (P = 0.0015, Figure 1A) and SAMD4A (P < 0.0001, Figure 1B) were significantly reduced in PBMCs from untreated CHB patients. These findings suggest that chronic HBV infection is associated with decreased MX2 and SAMD4A expression in PBMCs.

### Comparison of patient characteristics between responders and non-responders during Peg-IFN-α therapy

**FIGURE 2:**
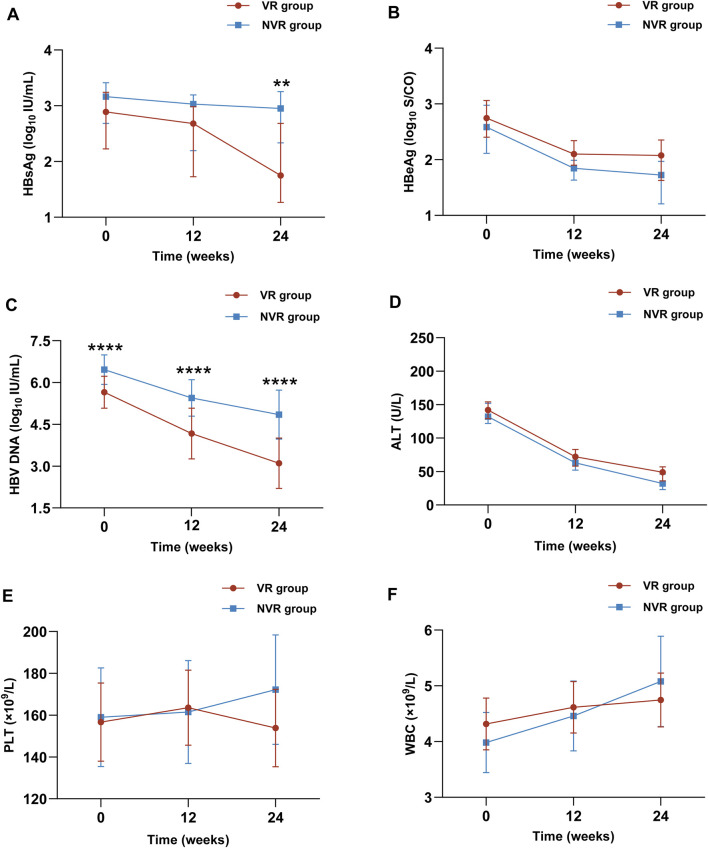
The time change of various clinical characteristics in the VR and NVR groups. HBsAg **(A)**, HBeAg **(B)**, HBV DNA **(C)**, ALT **(D)**, PLT **(E)**, and WBC **(F)** levels in VR and NVR patients receiving 48-week Peg-IFN-α therapy; *P < 0.05, **P < 0.01, ***P < 0.001, ****P < 0.0001; ●, represents the median value of each parameter in the group with virological response; ■, represents the median value of each parameter in the group without virological response; the bottom and top of the whiskers represent 25 and 75 percentiles of each parameter.

**FIGURE 3:**
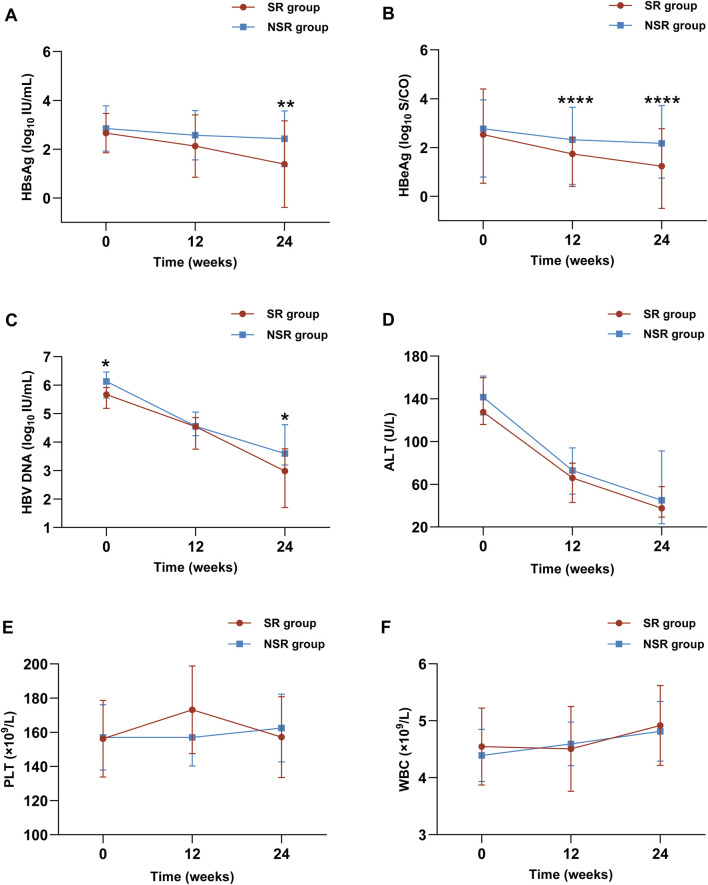
The time change of various clinical characteristics in the SR and NSR groups. HBsAg **(A)**, HBeAg **(B)**, HBV DNA **(C)**, ALT **(D)**, PLT **(E)**, and WBC **(F)** levels in SR and NSR patients receiving 48-week Peg-IFN-α therapy; *P < 0.05, **P < 0.01, ***P < 0.001, ****P < 0.0001; ●, represents the median value of each parameter in the group with serological response; ■, represents the median value of each parameter in the group without serological response; the bottom and top of the whiskers represent 25 and 75 percentiles of each parameter.

During Peg-IFN-α therapy, patients in the VR group exhibited significantly lower HBsAg levels than those in the NVR group at week 24 (P = 0.0034; Figure 2A; Supplementary Table S3). HBV DNA levels were also significantly lower in the VR group at weeks 0, 12, and 24 (all P < 0.0001; Figure 2C; Supplementary Table S3). No statistically significant differences were observed in HBeAg and ALT levels, or PLT and WBC counts between the two groups at weeks 0, 12, and 24 (Figure 2; Supplementary Table S3). Patients in the SR group exhibited significantly lower HBeAg levels than those in the NSR group at weeks 12 and 24 (all P < 0.0001; Figure 3B; Supplementary Table S4). HBsAg levels were reduced in the SR group at week 24 (P = 0.0087; Figure 3A; Supplementary Table S4). Furthermore, HBV DNA levels were significantly lower in the SR group than in the NSR group at week 0 (P = 0.0204) and at week 24 (P = 0.0261; Figure 3C; Supplementary Table S4). No statistically significant differences were found in ALT levels, or PLT and WBC counts between the two groups at weeks 0, 12, and 24 (Figure 3; Supplementary Table S4).

### Dynamic changes in MX2 and SAMD4A mRNA levels during early peg-IFN-α therapy in CHB patients

To determine the dynamic changes in ISGs during early therapy, we analyzed MX2 and SAMD4A mRNA levels in PBMCs at weeks 0, 12, and 24 in the responder and non-responder groups.

**FIGURE 4:**
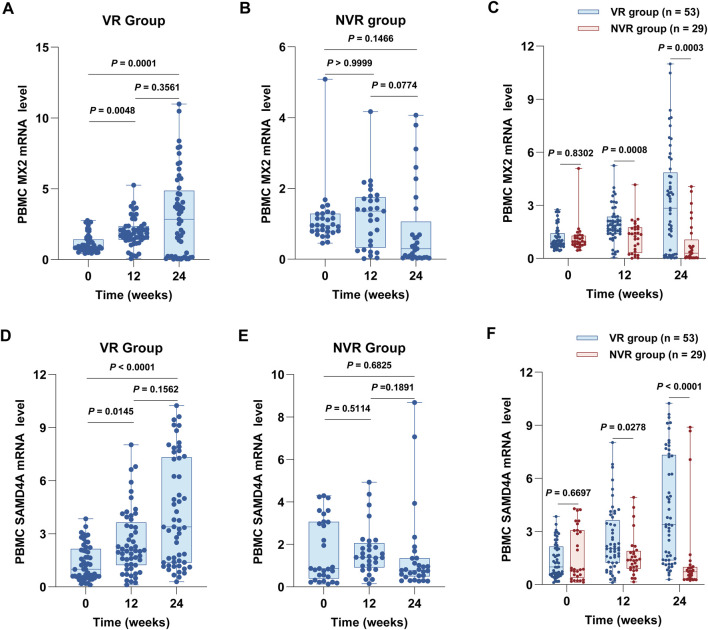
Dynamic changes of MX2 and SAMD4A mRNA levels in the VR and NVR groups. **(A,B)** MX2 mRNA levels during therapy in the VR group and the NVR group. **(D,E)** SAMD4A mRNA levels during therapy in the VR group and the NVR group. **(C,F)** MX2 and SAMD4A mRNA levels in the VR group and NVR group were compared at 0 weeks, 12 weeks and 24 weeks of therapy. MX2 and SAMD4A mRNA levels in PBMCs were measured by qRT-PCR. Values are described by median (interquartile range). P < 0.05 was statistically significant.

In the VR group, MX2 mRNA levels increased significantly at week 12 (P = 0.0048) and week 24 (P = 0.0001; Figure 4A). SAMD4A mRNA levels showed a similar increase at week 12 (P = 0.0145) and week 24 (P < 0.0001; Figure 4D). In contrast, MX2 mRNA levels showed no significant change in the NVR group at week 12 (P > 0.9999) or week 24 (P = 0.1466; Figure 4B). SAMD4A mRNA levels also showed no significant change at week 12 (P = 0.5114) and week 24 (P = 0.6825; Figure 4E). We next compared MX2 and SAMD4A mRNA levels between the VR and NVR groups. MX2 mRNA levels were significantly higher in the VR group than in the NVR group at both week 12 (P = 0.0008) and week 24 (P = 0.0003; Figure 4C). SAMD4A mRNA levels were significantly higher in the VR group than in the NVR group at week 12 (P = 0.0278) and week 24 (P < 0.0001; Figure 4F).

**FIGURE 5:**
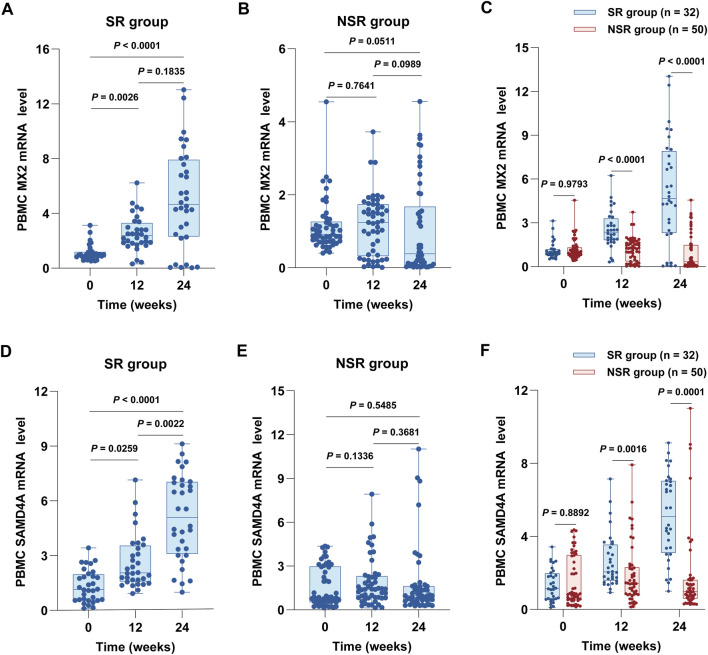
Dynamic changes of MX2 and SAMD4A mRNA levels in the SR and NSR groups. **(A,B)** MX2 mRNA levels during therapy in the SR group and the NSR group. **(D,E)** SAMD4A mRNA levels during therapy in the SR group and the NSR group. **(C,F)** MX2 and SAMD4A mRNA levels in the SR group and NSR group were compared at 0 weeks, 12 weeks and 24 weeks of therapy. MX2 and SAMD4A mRNA levels in PBMCs were measured by qRT-PCR. Values are described by median (interquartile range). P < 0.05 was statistically significant.

In the SR group, Peg-IFN-α therapy resulted in a significant increase in MX2 mRNA levels at week 12 (P = 0.0026) and week 24 (P < 0.0001; Figure 5A). SAMD4A mRNA levels also increased significantly at week 12 (P = 0.0259) and week 24 (P < 0.0001; Figure 5D). Conversely, Peg-IFN-α therapy did not lead to a significant increase in MX2 mRNA levels in the NSR group at week 12 (P = 0.7641) and week 24 (P = 0.0511; Figure 5B). SAMD4A mRNA levels also showed no significant change at week 12 (P = 0.1336) or week 24 (P = 0.5485; Figure 5E). We further compared MX2 and SAMD4A mRNA levels between the SR and NSR groups. MX2 mRNA levels were significantly higher in the SR group than in the NSR group at week 12 (P < 0.0001) and week 24 (P < 0.0001; Figure 5C). Similarly, SAMD4A mRNA levels were significantly higher in the SR group than in the NSR group at week 12 (P = 0.0016) and week 24 (P = 0.0001; Figure 5F).

Collectively, these results indicate distinct expression dynamics of MX2 and SAMD4A mRNA levels between the responder (VR/SR) and non-responder (NVR/NSR) groups during early Peg-IFN-α therapy, suggesting that higher MX2 and SAMD4A mRNA levels are associated with more favorable treatment outcomes.

### Correlations between MX2 and SAMD4A mRNA levels and reductions in HBeAg and HBV DNA during Peg-IFN-α therapy

**FIGURE 6:**
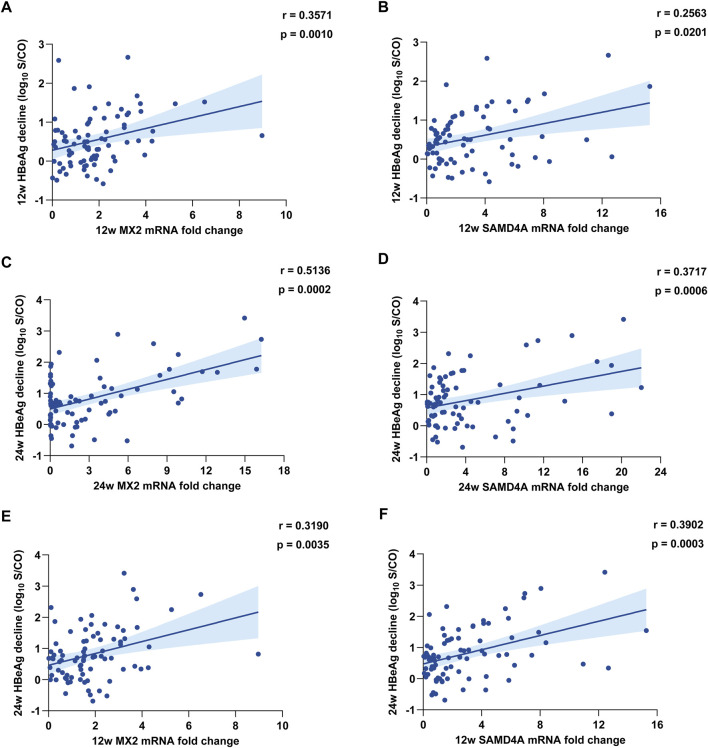
Correlation between MX2 and SAMD4A mRNA levels and HBeAg reduction during Peg-IFN-α therapy. **(A,C)** Correlation of MX2 mRNA levels with HBeAg reduction at weeks 12 and 24. **(B,D)** Correlation of SAMD4A mRNA levels with HBeAg reduction at weeks 12 and 24. **(E,F)** Correlation of MX2 and SAMD4A mRNA levels at week 12 with HBeAg reduction at week 24. MX2 and SAMD4A mRNA levels in PBMCs were quantified by qRT-PCR using the 2^−ΔΔCt^ method (Livak method), with GAPDH as the reference gene. HBeAg levels were log10 transformed prior to analysis. Correlations were assessed using Spearman’s rank correlation analysis. The correlation coefficient (r) and two-tailed P values were calculated. P < 0.05 was considered statistically significant.

We evaluated the associations between MX2 and SAMD4A mRNA levels and reductions in HBeAg and HBV DNA during therapy. At week 12, both MX2 (r = 0.3571, P = 0.0010; Figure 6A) and SAMD4A (r = 0.2563, P = 0.0201; Figure 6B) mRNA levels showed significant positive correlations with concurrent HBeAg reduction. Furthermore, these positive correlations persisted at week 24 for both MX2 (r = 0.5136, P = 0.0002; Figure 6C) and SAMD4A (r = 0.3717, P = 0.0006; Figure 6D). We next examined the temporal relationship between early gene expression and subsequent HBeAg reduction. Notably, MX2 (r = 0.3190, P = 0.0035; Figure 6E) and SAMD4A (r = 0.3902, P = 0.0003; Figure 6F) mRNA levels at week 12 were significantly correlated with HBeAg reduction at week 24.

**FIGURE 7:**
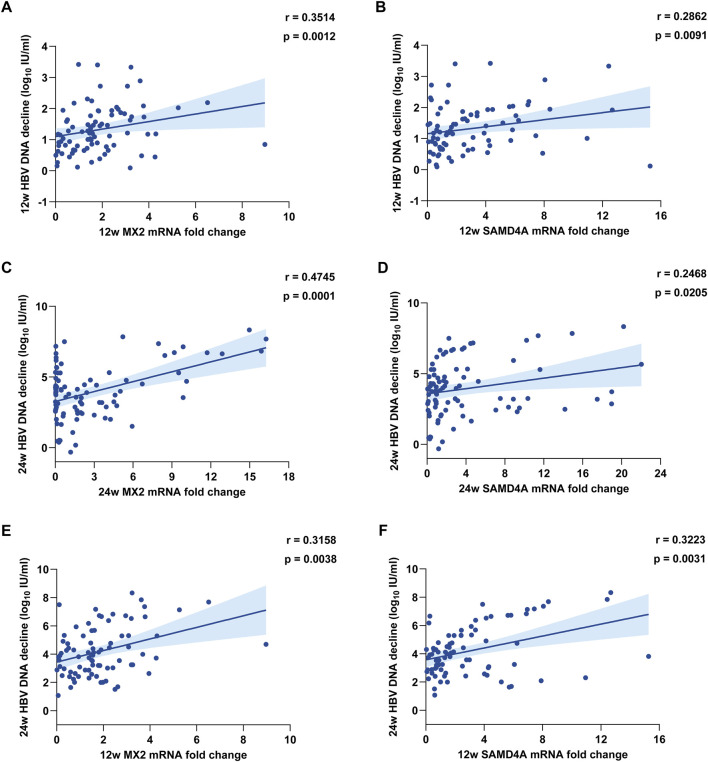
Correlation between MX2 and SAMD4A mRNA levels and HBV DNA reduction during Peg-IFN-α therapy. **(A,C)** Correlation of MX2 mRNA levels with HBV DNA reduction at weeks 12 and 24. **(B,D)** Correlation of SAMD4A mRNA levels with HBV DNA reduction at weeks 12 and 24. **(E,F)** Correlation of MX2 and SAMD4A mRNA levels at week 12 with HBV DNA reduction at week 24. MX2 and SAMD4A mRNA levels in PBMCs were quantified by qRT-PCR using the 2^−ΔΔCt^ method (Livak method), with GAPDH as the reference gene. HBV DNA levels were log10 transformed prior to analysis. Correlations were assessed using Spearman’s rank correlation analysis. The correlation coefficient (r) and two-tailed P values were calculated. P < 0.05 was considered statistically significant.

At week 12, both MX2 (r = 0.3514, P = 0.0012; Figure 7A) and SAMD4A (r = 0.2862, P = 0.0091; Figure 7B) mRNA levels showed significant positive correlations with concurrent HBV DNA reduction. These positive correlations remained significant at week 24 for both MX2 (r = 0.4745, P = 0.0001; Figure 7C) and SAMD4A (r = 0.2468, P = 0.0205; Figure 7D). We further examined the temporal relationship between early gene expression and subsequent HBV DNA reduction. Notably, MX2 (r = 0.3158, P = 0.0038; Figure 7E) and SAMD4A (r = 0.3223, P = 0.0031; Figure 7F) mRNA levels at week 12 were significantly correlated with HBV DNA reduction at week 24.

### Factors associated with virological or serological response to Peg-IFN-α therapy

Univariate analyses showed that HBV DNA at week 0 was significantly associated with VR, and at week 12, HBV DNA, MX2, and SAMD4A were also significantly associated with VR. At week 24, HBsAg, HBV DNA, MX2, and SAMD4A were significantly associated with VR (Supplementary Table S5). Multivariate analyses identified HBV DNA at week 0 as an independent predictor of VR, with an adjusted odds ratio (aOR) of 0.087 (P = 0.0001). At week 12, HBV DNA (aOR = 0.095, P < 0.0001), MX2 (aOR = 2.699, P = 0.0025), and SAMD4A (aOR = 1.770, P = 0.0104) were independent predictors of VR. At week 24, HBV DNA (aOR = 0.193, P = 0.0006), MX2 (aOR = 1.777, P = 0.0052), and SAMD4A (aOR = 1.604, P = 0.0008) remained independent predictors of VR.

Univariate analyses also indicated that HBV DNA was significantly associated with SR at week 0. At week 12, HBeAg, MX2, and SAMD4A were significantly associated with SR, and these associations remained significant at week 24 (Supplementary Table S6). Multivariate analyses revealed that HBV DNA (aOR = 0.484, P = 0.0485) at week 0 was an independent predictor of SR. At week 12, HBeAg (aOR = 0.143, P = 0.0020), MX2 (aOR = 3.654, P = 0.0008), and SAMD4A (aOR = 2.033, P = 0.0012) were independent predictors of SR, and at week 24, HBeAg (aOR = 0.141, P = 0.0068), MX2 (aOR = 2.255, P = 0.0016), and SAMD4A (aOR = 1.730, P = 0.0003) remained independent predictors of SR (Supplementary Table S6).

### Predictive values of MX2 and SAMD4A mRNA levels for VR and SR

We further evaluated the predictive performance of MX2 and SAMD4A mRNA levels in PBMCs for discriminating responses to Peg-IFN-α therapy in HBeAg-positive CHB patients. ROC curve analyses were conducted to assess the predictive capabilities of MX2 and SAMD4A mRNA levels at weeks 0, 12, and 24 for VR and SR.

**FIGURE 8:**
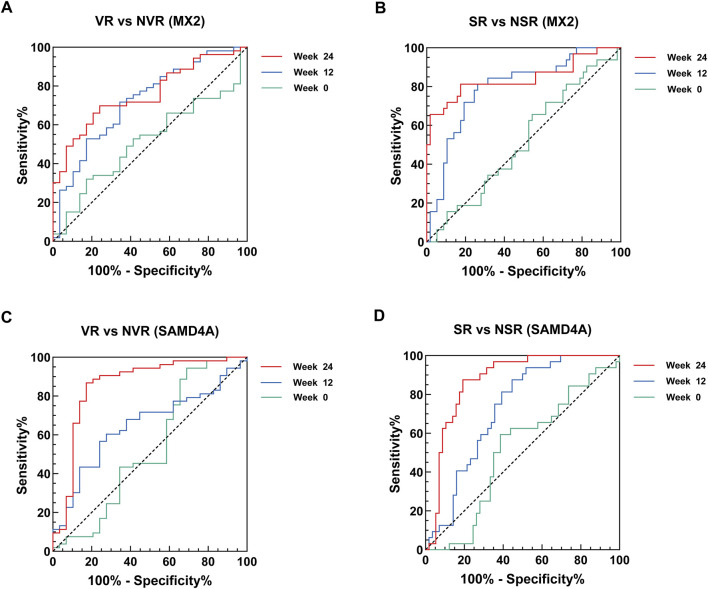
Receiver operating characteristic (ROC) curves for predicting VR and SR after 48 weeks of Peg-IFN-α therapy. **(A,B)** ROC curves of MX2 at weeks 0, 12, and 24 for predicting VR and SR. **(C,D)** ROC curves of SAMD4A at weeks 0, 12, and 24 for predicting VR and SR. The Youden index was used to maximize the potential effectiveness of the biomarkers.

MX2 mRNA levels at week 24 demonstrated the highest predictive accuracy for VR, with an AUC of 0.7567 (P = 0.0001). The optimal cut-off value at week 24 was 0.9785, yielding a sensitivity of 69.80% and a specificity of 78.90%, as determined using Youden’s index (Figure 8A; Supplementary Table S7). For SR prediction, MX2 mRNA levels at week 24 also exhibited the best predictive performance, with an AUC of 0.8421 (P = 0.0002). The optimal cut-off value was 3.906, corresponding to a sensitivity of 74.60% and a specificity of 98.00% (Figure 8B; Supplementary Table S7). For SAMD4A, mRNA levels at week 24 showed the strongest predictive performance for VR, with an AUC of 0.8549 (P < 0.0001). The optimal cut-off value was 1.1151, providing a sensitivity of 86.60% and a specificity of 82.80% (Figure 8C; Supplementary Table S7). Furthermore, SAMD4A mRNA levels at week 24 demonstrated superior predictive capability for SR, with an AUC of 0.8717 (P = 0.0003). The optimal cut-off value was 2.0554, achieving a sensitivity of 87.50% and a specificity of 84.00% (Figure 8D; Supplementary Table S7).

### Comparison of the predictive performance of MX2 and SAMD4A with conventional viral kinetic markers

To compare the predictive performance of MX2 and SAMD4A with conventional viral kinetic markers, their individual discriminative abilities were evaluated against HBsAg decline. For VR prediction, MX2 at week 12 demonstrated predictive performance comparable to HBsAg decline (AUC = 0.7274; P = 0.8644), but exhibited a statistically significant difference at week 24 compared with HBsAg decline (AUC = 0.8031; P = 0.0497). SAMD4A at week 12 exhibited a statistically significant difference compared with HBsAg decline (AUC = 0.7274; P = 0.0305), whereas SAMD4A achieved significantly higher predictive accuracy at week 24 than HBsAg decline (AUC = 0.8031; P = 0.0316; Supplementary Figure S2; Supplementary Table S8). For SR prediction, MX2 at both week 12 and week 24 demonstrated significantly greater predictive performance than HBsAg decline (AUC = 0.6706, P = 0.0319; and AUC = 0.7292, P = 0.0032, respectively). Similarly, SAMD4A at week 12 and week 24 exhibited significantly higher predictive accuracy than HBsAg decline (AUC = 0.6706, P = 0.0480; and AUC = 0.7292, P = 0.0015, respectively; Supplementary Figure S2; Supplementary Table S8).

Furthermore, we also compared the predictive performance of MX2 and SAMD4A with HBV DNA decline. For VR prediction, MX2 at week 12 and week 24 showed predictive performance comparable to HBV DNA decline (AUC = 0.6845, P = 0.2098; and AUC = 0.7310, P = 0.2719, respectively). Similarly, SAMD4A at week 12 showed no significant difference compared with HBV DNA decline (AUC = 0.6845; P = 0.3246), whereas significantly greater predictive performance was observed for SAMD4A at week 24 compared with HBV DNA decline (AUC = 0.7310; P = 0.0251; Supplementary Figure S3; Supplementary Table S9). For SR prediction, both MX2 and SAMD4A demonstrated significantly higher predictive accuracy than HBV DNA decline at weeks 12 and 24. Specifically, MX2 at week 12 and week 24 showed significantly greater predictive performance than HBV DNA decline (AUC = 0.6381, P = 0.0137; and AUC = 0.8138, P = 0.0418, respectively). Finally, SAMD4A at week 12 and week 24 also exhibited significantly higher predictive accuracy than HBV DNA decline (AUC = 0.6381, P = 0.0281; and AUC = 0.8138, P = 0.0152, respectively; Supplementary Figure S3; Supplementary Table S9). These findings suggest that MX2 and SAMD4A may provide complementary predictive information beyond conventional viral kinetic markers during Peg-IFN-α therapy.

### Internal validation of MX2 and SAMD4A predictive models for VR and SR to Peg-IFN-α therapy

**FIGURE 9:**
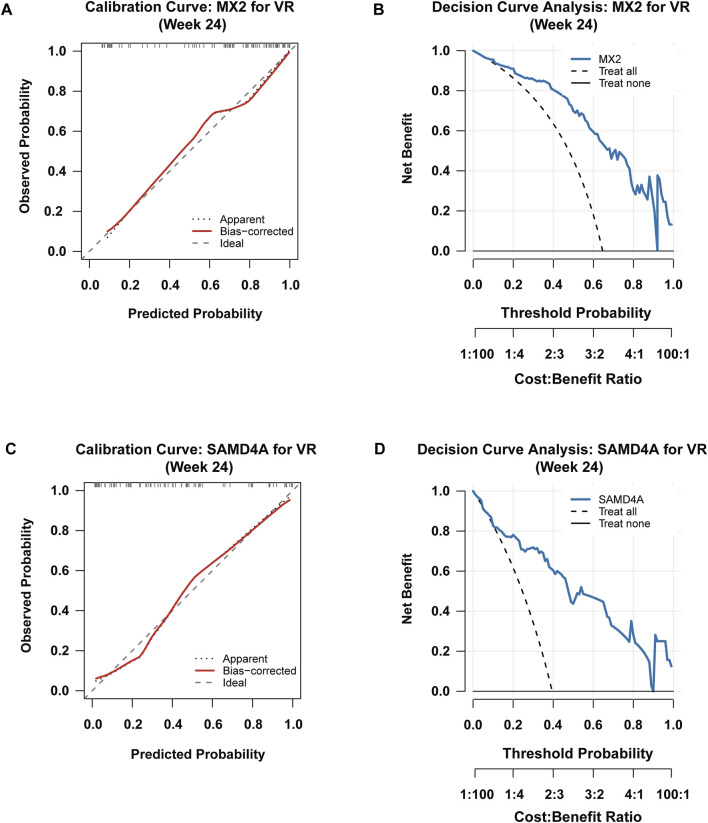
Internal validation and clinical utility of MX2 and SAMD4A in predicting VR. **(A)** Calibration curve and **(B)** DCA evaluating the predictive performance and net clinical benefit of MX2 mRNA levels at week 24. **(C)** Calibration curve and **(D)** DCA evaluating the predictive performance and net clinical benefit of SAMD4A mRNA levels at week 24. Calibration curves were plotted using bootstrap resampling with 1,000 iterations to estimate optimism-corrected performance.

To further assess the stability of the predictive models, internal validation was performed for the models at week 24 using bootstrap resampling with 1,000 iterations, together with calibration curve analysis and DCA. For VR prediction, the optimism-corrected C-index was 0.741 for MX2 and 0.845 for SAMD4A. Calibration analysis showed good agreement between predicted and observed probabilities, with calibration slopes of 0.963 and 0.979, intercepts of 0.001 and 0.008, and Brier scores of 0.133 and 0.114 for MX2 and SAMD4A, respectively (Supplementary Table S10). The calibration curves demonstrated close agreement between the apparent and bias-corrected estimates and the ideal reference line (Figures 9A,C). DCA further showed favorable net benefit across a broad range of threshold probabilities for both models (Figures 9B,D).

**FIGURE 10:**
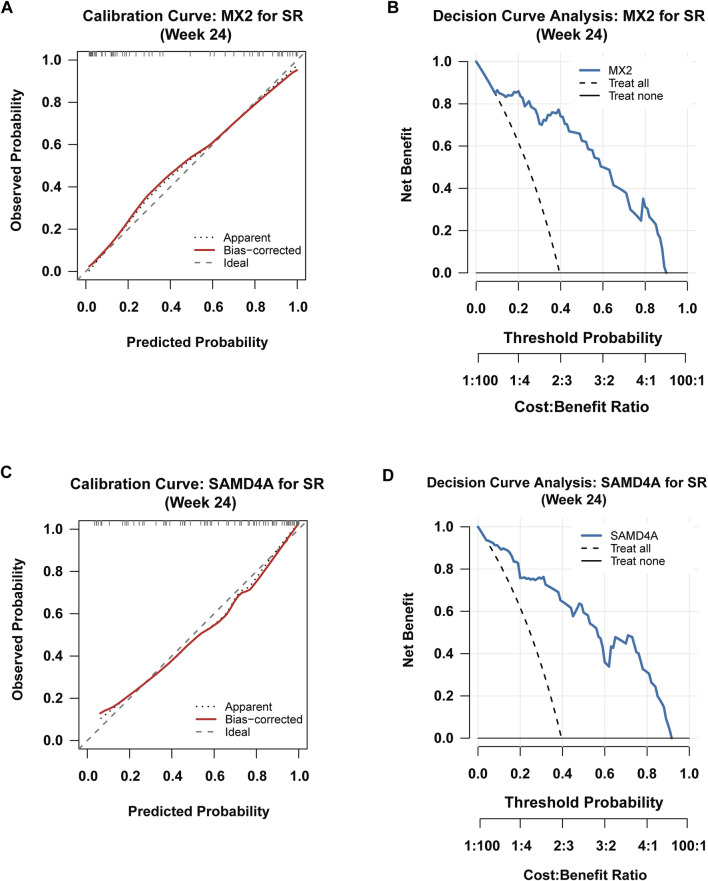
Internal validation and clinical utility of MX2 and SAMD4A in predicting SR. **(A)** Calibration curve and **(B)** DCA illustrating the predictive accuracy and net benefit of MX2 mRNA levels at week 24. **(C)** Calibration curve and **(D)** DCA illustrating the predictive accuracy and net benefit of SAMD4A mRNA levels at week 24. Calibration curves were plotted using bootstrap resampling with 1,000 iterations to estimate optimism-corrected performance.

For SR prediction, the optimism-corrected C-index was 0.835 for MX2 and 0.863 for SAMD4A. Calibration slopes were 0.946 and 0.988, with intercepts of 0.003 and 0.009, and Brier scores of 0.126 and 0.103, respectively (Supplementary Table S11). The calibration curves similarly demonstrated good agreement between predicted and observed probabilities (Figures 10A,C). In addition, DCA indicated favorable net benefit across clinically relevant threshold probabilities for both models (Figures 10B,D).

Overall, the internal validation analyses demonstrated that the predictive models based on MX2 and SAMD4A at week 24 maintained stable discriminative performance, good calibration, and potential clinical utility within the current cohort.

## Discussion

Peg-IFN-α remains one of the two first-line therapeutic options recommended by current clinical practice guidelines for patients with CHB (Wong and Lemoine, 2025; EASL, 2025). Although Peg-IFN-α–based strategies can achieve functional cure rates exceeding 30% in selected populations, a substantial proportion of patients fail to achieve HBsAg clearance, highlighting the need for reliable predictors of treatment response (Chu et al., 2022; Li et al., 2024; Yang et al., 2022). Early identification of patients likely to benefit from therapy would improve treatment stratification and minimize unnecessary exposure to interferon-related adverse effects. Peg-IFN-α exerts potent antiviral effects primarily through the induction of ISGs and the modulation of host immune responses (Schoggins et al., 2011; Li et al., 2022). Previous studies have demonstrated that gene expression profiling in PBMCs may provide valuable information for predicting disease progression and treatment response in CHB. For example, IFI16 has been shown to sense HBV infection and regulate antiviral immunity (Lu et al., 2021), while classical ISGs such as STAT1 and MX1 are closely involved in antiviral defense (Han et al., 2017). However, the exact clinical relevance of MX2 and SAMD4A in HBV infection and interferon response remains insufficiently characterized.

In the present study, we demonstrate that MX2 and SAMD4A expression levels in PBMCs are associated with antiviral response to Peg-IFN-α therapy. We longitudinally analyzed the dynamic changes in MX2 and SAMD4A expression alongside clinical parameters, including HBsAg, HBeAg, HBV DNA, ALT, PLT, and WBC, in HBeAg-positive CHB patients receiving 48 weeks of treatment. Based on our results, it can be suggested that MX2 and SAMD4A may be considered as promising candidates for on-treatment biomarkers. Previous studies have reported that MX2 and SAMD4A can be induced by type I interferon and exhibit antiviral activity against HBV (Wang et al., 2020; Wang et al., 2021; Schoggins et al., 2011). Therefore, we first compared MX2 and SAMD4A mRNA levels in PBMCs from healthy controls and untreated CHB patients and found that untreated CHB patients had significantly lower baseline levels of MX2 and SAMD4A expression compared with healthy controls, suggesting that chronic HBV infection is associated with decreased expression of these genes in PBMCs.

Although HBsAg loss is considered the optimal endpoint for functional cure, its occurrence remains rare in clinical practice. HBeAg seroconversion is therefore widely used as a surrogate endpoint for evaluating treatment efficacy (Terrault et al., 2018; EASL, 2025; Dienstag, 2008). In our cohort, 53 patients (64.63%) achieved the VR and 32 patients (39.02%) achieved the SR after 48 weeks of therapy, rates comparable to those reported in previous studies on Peg-IFN-α therapy (Fried et al., 2008; Lau et al., 2005; Zhu et al., 2018). Several clinical parameters, including ALT, HBsAg, HBeAg, HBV DNA, and viral genotype, have been associated with HBeAg seroconversion in HBeAg-positive CHB patients receiving Peg-IFN-α therapy (ter Borg et al., 2008; Fried et al., 2008; Moucari et al., 2009b). In our study, we monitored the serum kinetics of HBsAg, HBeAg, HBV DNA, ALT, PLT, and WBC at various time points during early treatment. HBsAg levels decreased gradually over time, reflecting the difficulty of achieving complete HBsAg clearance, which is closely related to the persistence of intrahepatic cccDNA (Bae et al., 2025; Gao et al., 2017). In contrast, HBeAg and HBV DNA exhibited a rapid decline following the initiation of treatment, reflecting effective suppression of viral replication. Patients who responded to therapy exhibited a greater reduction in HBsAg and HBV DNA levels than non-responders, indicating a more efficient antiviral effect of interferon in these patients. Further analyses showed that HBV DNA was an independent predictor of both VR and SR. Given that previous studies have reported inconsistent associations between conventional clinical parameters and treatment outcomes (Sonneveld et al., 2012; Rijckborst et al., 2010; Sonneveld et al., 2013), our findings underscore the need to identify additional biomarkers that better reflect host antiviral immunity.

Previous studies have demonstrated a strong relationship between ISGs and the antiviral immune response (Allweiss et al., 2014; Hou et al., 2014; Fletcher et al., 2015). However, substantial variability in treatment response remains, making the prediction of Peg-IFN-α efficacy particularly important. In our study, responders to Peg-IFN-α exhibited a more pronounced decline in HBsAg throughout the treatment period compared to non-responders. Based on this observation, we further examined whether differential ISG activation contributes to the response to Peg-IFN-α therapy. We found that the dynamic changes in the mRNA levels of MX2 and SAMD4A in PBMCs differed between the responder and non-responder groups. Notably, responders exhibited significantly higher MX2 and SAMD4A mRNA levels during early treatment, particularly at weeks 12 and 24. Given their direct anti-HBV activity, increased MX2 and SAMD4A expression during Peg-IFN-α therapy may reflect more effective activation of interferon-mediated antiviral immunity and a stronger host antiviral response associated with improved treatment outcomes. Therefore, the expression and activation of these genes may predict the antiviral effects of Peg-IFN-α therapy. Moreover, higher expression levels in responders suggest that early and sustained activation of these ISGs may be associated with improved viral control. Our correlation analyses provide further insight into the temporal dynamics between ISG expression and viral antigen decline during Peg-IFN-α therapy. The significant positive correlations observed between MX2 and SAMD4A mRNA levels and reductions in HBeAg and HBV DNA at both weeks 12 and 24 suggest that sustained ISG activation is closely associated with ongoing antigen clearance. Time-lagged analyses further showed that expression levels of MX2 and SAMD4A at week 12 were associated with subsequent reductions in HBeAg and HBV DNA at week 24, indicating that early immune activation may precede and predict later virological outcomes.

Recent studies have shown that MX2 can inhibit HCV replication (Yi et al., 2019), and polymorphisms of MX2 have been associated with biochemical indices and viral subtypes in patients with HCV (He et al., 2023). In addition, SAMD4A expression has been linked to host susceptibility to HBV infection and is negatively correlated with viral load (Wang et al., 2021). In our study, both univariate and multivariate analyses identified MX2 and SAMD4A expression at weeks 12 and 24 as independent predictors of treatment response, further supporting their clinical relevance. Interestingly, HBV DNA also demonstrated significant predictive value for SR and VR, which is consistent with findings from previous studies (Zhang et al., 2020; Wu et al., 2020). Using ROC curve analyses, we further evaluated the predictive performance of MX2 and SAMD4A. Both markers showed the highest predictive accuracy at week 24, indicating that their expression levels during the early stages of Peg-IFN-α therapy may be associated with improved therapeutic efficacy and prognosis. The superior predictive performance at week 24 emerged from an objective, data-driven comparison across all evaluated time points. Biologically, Peg-IFN-α–induced immune remodeling is time-dependent, and earlier on-treatment time points may represent a transitional phase of immune activation with greater inter-individual variability (Schoggins, 2014). Because both viral response kinetics and interferon-stimulated immune activation evolve progressively during therapy, later time points may better reflect the integrated host antiviral immune status. Accordingly, the sustained upregulation of MX2 and SAMD4A at week 24 may provide more robust discrimination of 48-week treatment outcomes than earlier phases. These findings are also consistent with the clinical importance of week 24 as an on-treatment evaluation time point during Peg-IFN-α therapy (Terrault et al., 2018; Kong et al., 2025).

To further evaluate the clinical utility of MX2 and SAMD4A, we compared their predictive performance with conventional viral kinetic markers, including HBsAg decline and HBV DNA decline. Interestingly, both ISGs performed comparably to conventional viral kinetic markers and demonstrated significantly greater predictive performance in several analyses of SR. These findings suggest that host ISGs may capture aspects of antiviral immune activation that are not fully reflected by viral kinetics alone. Importantly, host-response biomarkers were evaluated in the context of established virological markers, with the aim of assessing their potential complementary clinical relevance rather than replacing conventional predictors. While HBsAg and HBV DNA kinetics primarily reflect viral transcriptional activity and viral burden (Cornberg et al., 2017), MX2 and SAMD4A may partially reflect interferon-induced host antiviral immune activation. Therefore, these markers may serve complementary roles in treatment monitoring and response prediction. Although HBV DNA levels showed significant predictive ability in our study, MX2 and SAMD4A may provide complementary value for further investigation and clinical application. Future studies can investigate whether the combination of these biomarkers provides a more comprehensive approach to predicting treatment outcomes, particularly when HBV DNA alone does not fully reflect immune dynamics or viral persistence. This could guide clinicians in selecting appropriate treatment strategies and adjusting therapy promptly to minimize side effects and reduce medical costs. Additionally, although we did not measure other classical ISGs in our cohort, previous studies have reported their predictive performance for Peg-IFN-α therapy. For instance, circulating IP-10 showed an AUC of 0.723 for predicting viral clearance (Kan et al., 2024), Mx1 induction exhibited an AUC of 0.838 for predicting sustained virologic responses to IFN-α (Kong et al., 2007), and PBMC-derived TUBB1 promoter methylation demonstrated an AUC of 0.805 for HBeAg seroconversion (Zhao et al., 2025). Notably, MX2 and SAMD4A in our study exhibited AUCs of 0.7567–0.8717, suggesting potentially comparable predictive performance across different clinical settings. These comparisons highlight the potential complementary value of MX2 and SAMD4A as early on-treatment biomarkers, suggesting that their measurement in PBMCs could provide additional insight into host antiviral responses and support improved patient stratification for Peg-IFN-α therapy. In addition, we performed bootstrap-based internal validation to further evaluate the stability of the predictive models within the current cohort. The close agreement between the apparent and bias-corrected calibration curves, together with the favorable discrimination and the net clinical benefit demonstrated by DCA, suggests that the predictive performance of MX2 and SAMD4A was not solely attributable to model overfitting. These findings provide additional support for the robustness of the observed associations and the potential clinical applicability of these ISGs as on-treatment biomarkers during Peg-IFN-α therapy.

However, several limitations should be acknowledged in this study. First, the duration of follow-up was relatively short, limiting the assessment of sustained response post-treatment. Second, serum HBV RNA, which has recently emerged as a promising biomarker reflecting intrahepatic cccDNA transcriptional activity and interferon responsiveness (Giersch et al., 2017; van Bömmel et al., 2018), was not routinely measured in the current cohort. Consequently, direct comparisons between MX2/SAMD4A and HBV RNA kinetics could not be performed. Further investigations incorporating HBV RNA measurements are needed to clarify the incremental clinical value of these ISGs during Peg-IFN-α therapy. Third, most patients were infected with HBV genotypes B or C, which may influence interferon responsiveness, as genotype B has been associated with better treatment outcomes than genotype C (Wai et al., 2002; Kao et al., 2000). In addition, the present cohort primarily consisted of Asian patients, which may further limit the broader applicability of our findings to non-Asian populations or to patients infected with other HBV genotypes. Nevertheless, MX2 and SAMD4A are host ISGs that reflect antiviral immune activation rather than genotype-specific viral characteristics. Although treatment responsiveness may vary across HBV genotypes and ethnic populations, the biological relevance of these ISGs as host-response biomarkers may still extend across different clinical settings. Additional studies involving more diverse ethnic backgrounds and HBV genotypes are therefore warranted to further evaluate the performance of these biomarkers across different populations. Fourth, the sample size was relatively small and only HBeAg-positive patients were included, excluding HBeAg-negative CHB patients, which may limit the generalizability of our findings. Evaluation of early treatment responses in HBeAg-negative CHB patients presents additional challenges because this population lacks HBeAg seroconversion as a conventional treatment endpoint and often exhibits distinct virological characteristics and more heterogeneous interferon-response kinetics, which may require dedicated cohort designs for biomarker validation (Islam et al., 2023; Zhang et al., 2026; Hadziyannis and Laras, 2018). Finally, the present study was conducted in a single-center prospective cohort without an independent external validation cohort. Although internal validation supported the overall consistency of the predictive models, external validation in larger multicenter populations remains necessary to further confirm the clinical utility of MX2 and SAMD4A during Peg-IFN-α therapy.

## Conclusion

Our study demonstrates that increased expression of MX2 and SAMD4A in PBMCs is associated with improved early response to Peg-IFN-α therapy in HBeAg-positive CHB patients. Therefore, MX2 and SAMD4A could serve as early biomarkers for enhancing the management of interferon therapy in HBV-infected patients.

## Data Availability

The raw data supporting the conclusions of this article will be made available by the authors, without undue reservation.
