# Supplementary material for: Altered expression of MX2 and SAMD4A in PBMCs predicts early treatment responses in HBeAg-positive chronic hepatitis B patients during Peg-IFN-α therapy
Source: Front Pharmacol. 2026 Jun 22;17:1844257. doi: 10.3389/fphar.2026.1844257 (PMC13333471; doi:10.3389/fphar.2026.1844257)
Supplement: Supplementary file 1 [file Table1.docx]

| **Table S1** Primer sequences used in this study | |
| --- | --- |
| Primers sequence(5’-3’) | |
| GAPDH RP | 5’-TGACACGTTGGCAGTGG-3’ |
| GAPDH FP | 5’-GGGGCTCTCCAGAACATC-3’ |
| MX2 RP | 5’-TGTGTGGACCCCTGATG-3’ |
| MX2 FP | 5’-TGGTTGTTTTGTGCGT-3’ |
| SAMD4A RP | 5’- CTGGACTTGCTGACTGACC -3’ |
| SAMD4A FP | 5’-AGGGAGAGGAGGGGAGA-3’ |
