# Supplementary material for: Altered expression of MX2 and SAMD4A in PBMCs predicts early treatment responses in HBeAg-positive chronic hepatitis B patients during Peg-IFN-α therapy
Source: Front Pharmacol. 2026 Jun 22;17:1844257. doi: 10.3389/fphar.2026.1844257 (PMC13333471; doi:10.3389/fphar.2026.1844257)
Supplement: Supplementary file 6 [file Table7.docx]

| **Table S7** The predictive performance of MX2 and SAMD4A mRNA levels in predicting VR and SR to 48 weeks of Peg-IFN-α treatment. | | | | | | | | | | | | |
| --- | --- | --- | --- | --- | --- | --- | --- | --- | --- | --- | --- | --- |
|  |  | MX2 |  | SAMD4A |  | MX2 |  | SAMD4A |  | MX2 |  | SAMD4A |
| VR prediction |  | **Week0** | | |  | **Week12** | | |  | **Week24** | | |
|  | AUC | 0.5146 |  | 0.5290 |  | 0.7209 |  | 0.6474 |  | 0.7567 |  | 0.8549 |
|  | (95% CI) | (0.3875 -   0.6418) |  | (0.3842 -  0.6737) |  | (0.6052 - 0.8366) |  | (0.5263 -  0.7685) |  | (0.6534 - 0.8599) |  | (0.7553 -  0.9545) |
|  | Cut-off value | 0.7433 |  | 2.9719 |  | 1.4847 |  | 1.8603 |  | 0.9785 |  | 1.1151 |
|  | Sensitivity (%) | 32.10 |  | 94.30 |  | 71.70 |  | 60.40 |  | 69.80 |  | 86.60 |
|  | Specificity (%) | 82.80 |  | 31.00 |  | 65.50 |  | 72.40 |  | 78.90 |  | 82.80 |
|  | P-value | 0.8273 |  | 0.6660 |  | **0.0010** |  | **0.0280** |  | **0.0001** |  | **<0.0001** |
| SR prediction |  | **Week0** | | |  | **Week12** | | |  | **Week24** | | |
|  | AUC | 0.5148 |  | 0.5115 |  | 0.7933 |  | 0.7249 |  | 0.8421 |  | 0.8717 |
|  | (95% CI) | (0.3912 -  0.6384) |  | (0.3884 -  0.6346) |  | (0.6948 - 0.8918) |  | (0.6210 -  0.8287) |  | (0.7425 - 0.9418) |  | (0.7967 -  0.9467) |
|  | Cut-off value | 0.7741 |  | 2.8296 |  | 1.7587 |  | 1.4173 |  | 3.906 |  | 2.0554 |
|  | Sensitivity (%) | 81.30 |  | 96.90 |  | 71.40 |  | 87.50 |  | 74.60 |  | 87.50 |
|  | Specificity (%) | 32.00 |  | 28.00 |  | 80.50 |  | 52.00 |  | 98.00 |  | 84.00 |
|  | P-value | 0.8174 |  | 0.8575 |  | **<0.0001** |  | **0.0005** |  | **0.0002** |  | **0.0003** |
| MX2, Myxovirus resistance 2; SAMD4A, Sterile alpha motif domain-containing 4A; AUC, area under ROC curve; CI, confidence interval; VR, virological response; SR, serological response; Bold values are statistically significant P < 0.05. | | | | | | | | | | | | |
