# Supplementary material for: Altered expression of MX2 and SAMD4A in PBMCs predicts early treatment responses in HBeAg-positive chronic hepatitis B patients during Peg-IFN-α therapy
Source: Front Pharmacol. 2026 Jun 22;17:1844257. doi: 10.3389/fphar.2026.1844257 (PMC13333471; doi:10.3389/fphar.2026.1844257)
Supplement: Supplementary file 8 [file Table9.docx]

| **Table S9** Comparison of predictive performance of MX2 and SAMD4A with HBV DNA decline at week 12 and 24 for predicting VR and SR. | | | | | | | | |
| --- | --- | --- | --- | --- | --- | --- | --- | --- |
| Response prediction |  | MX2  (week 12) | SAMD4A  (week 12) | HBV DNA decline (week 12) |  | MX2  (week 24) | SAMD4A  (week 24) | HBV DNA decline (week 24) |
| VR prediction | AUC | 0.7209 | 0.6474 | 0.6845 |  | 0.7567 | 0.8549 | 0.7310 |
|  | (95% CI) | (0.6052 - 0.8366) | (0.5263 - 0.7685) | (0.5600 - 0.8089) |  | (0.6534 - 0.8599) | (0.7553 - 0.9545) | (0.6225 - 0.8375) |
|  | Cut-off value | 1.4847 | 1.8603 | 1.334 |  | 0.9785 | 1.1151 | 4.1288 |
|  | Sensitivity (%) | 71.70 | 60.40 | 75.50 |  | 69.80 | 86.60 | 62.30 |
|  | Specificity (%) | 65.50 | 72.40 | 62.10 |  | 78.90 | 82.80 | 89.70 |
|  | P value (DeLong's test, vs. HBV DNA decline) | 0.2098 | 0.3246 |  |  | 0.2719 | **0.0251** |  |
| SR prediction | AUC | 0.7933 | 0.7249 | 0.6381 |  | 0.8421 | 0.8717 | 0.8138 |
|  | (95% CI) | (0.6948 - 0.8918) | (0.6210 - 0.8287) | (0.5122 - 0.7641) |  | (0.7425 - 0.9418) | (0.7967 - 0.9467) | (0.7124 - 0.9151) |
|  | Cut-off value | 1.7587 | 1.4173 | 1.1037 |  | 3.906 | 2.0554 | 4.4397 |
|  | Sensitivity (%) | 71.40 | 87.50 | 75.00 |  | 74.60 | 87.50 | 71.90 |
|  | Specificity (%) | 80.50 | 52.00 | 54.00 |  | 98.00 | 84.00 | 86.50 |
|  | P value (DeLong's test, vs. HBV DNA decline) | **0.0137** | **0.0281** |  |  | **0.0418** | **0.0152** |  |
| MX2, Myxovirus resistance 2; SAMD4A, Sterile alpha motif domain-containing 4A; AUC, area under ROC curve; CI, confidence interval; VR, virological response; SR, serological response; Bold values are statistically significant P < 0.05. | | | | | | | | |
