# Supplementary material for: Altered expression of MX2 and SAMD4A in PBMCs predicts early treatment responses in HBeAg-positive chronic hepatitis B patients during Peg-IFN-α therapy
Source: Front Pharmacol. 2026 Jun 22;17:1844257. doi: 10.3389/fphar.2026.1844257 (PMC13333471; doi:10.3389/fphar.2026.1844257)
Supplement: Supplementary file 9 [file Table2.docx]

| **Table S2** Treatment responses during Peg-IFN-α treatment | | | | |
| --- | --- | --- | --- | --- |
| Treatment responses (n, %) | 12w | 24w | 36w | 48w |
| Virological response | 3(3.66%) | 13(15.85%) | 37(45.12%) | 53(64.63%) |
| Serological response | 0 (0.00%) | 4 (4.88%) | 11 (13.41%) | 32(39.02%) |
