# Supplementary material for: Altered expression of MX2 and SAMD4A in PBMCs predicts early treatment responses in HBeAg-positive chronic hepatitis B patients during Peg-IFN-α therapy
Source: Front Pharmacol. 2026 Jun 22;17:1844257. doi: 10.3389/fphar.2026.1844257 (PMC13333471; doi:10.3389/fphar.2026.1844257)
Supplement: Supplementary file 10 [file Table5.docx]

| **Table S5** On-treatment variables associated with virological response according to univariate and multivariate analyses | | | | | | | | | | | | |
| --- | --- | --- | --- | --- | --- | --- | --- | --- | --- | --- | --- | --- |
| Variables | Univariate analyses |  | Multivariate analyses |  | Univariate analyses |  | Multivariate analyses |  | Univariate analyses |  | Multivariate analyses |  |
|  | OR(95%CI) | P-value | aOR(95%CI) | P-value | OR(95%CI) | P- value | aOR(95%CI) | P-value | OR(95%CI) | P-value | aOR(95%CI) | P-  value |
|  | **Week0** |  |  |  | **Week12** |  |  |  | **Week24** |  |  |  |
| Gender | 1.977(0.784, 4.985) | 0.1484 |  |  | 1.977(0.784, 4.985) | 0.1484 |  |  | 1.977(0.784, 4.985) | 0.1484 |  |  |
| Age | 0.972(0.929, 1.016) | 0.2174 |  |  | 0.972(0.929, 1.016) | 0.2174 |  |  | 0.972(0.929, 1.016) | 0.2174 |  |  |
| HBsAg | 0.774(0.452, 1.326) | 0.0918 |  |  | 0.619(0.382, 1.004) | 0.0522 |  |  | 0.545(0.349, 0.852) | **0.0077** | 0.678(0.342  , 1.345) | 0.2673 |
| HBV DNA | 0.103(0.038, 0.279) | **0.0001** | 0.087(0.029,0.256) | **0.0001** | 0.096(0.035, 0.262) | **0.0001** | 0.095(0.030  , 0.298) | **<0.0001** | 0.130(0.055, 0.303) | **<0.0001** | 0.193(0.075  , 0.497) | **0.0006** |
| HBeAg | 1.443(0.789, 2.637) | 0.2330 |  |  | 1.581(0.828, 3.018) | 0.1643 |  |  | 1.340(0.805, 2.232) | 0.2594 |  |  |
| ALT | 1.007(0.991, 1.024) | 0.3702 |  |  | 1.009(0.991, 1.010) | 0.8360 |  |  | 1.005(0.995, 1.016) | 0.2751 |  |  |
| WBC | 0.877(0.672, 1.144) | 0.8770 |  |  | 1.059(0.801, 1.401) | 0.6827 |  |  | 0.882(0.692, 1.123) | 0.3094 |  |  |
| PLT | 0.997(0.993, 1.006) | 0.9935 |  |  | 1.090(0.993, 1.007) | 0.8524 |  |  | 0.995(0.988, 1.002) | 0.2386 |  |  |

**Continued Table S5** On-treatment variables associated with virological response according to univariate and multivariate analyses

| Variables | Univariate analyses |  | Multivariate analyses |  | Univariate analyses |  | Multivariate analyses |  | Univariate analyses |  | Multivariate analyses |  |
| --- | --- | --- | --- | --- | --- | --- | --- | --- | --- | --- | --- | --- |
|  | OR(95%CI) | P-value | aOR(95%CI) | P-value | OR(95%CI) | P-value | aOR(95%CI) | P-value | OR(95%CI) | P-value | aOR(95%CI) | P-  value |
| MX2 | 1.039(0.542, 1.993) | 0.9070 |  |  | 2.4361.344  , 4.417) | **0.0033** | 2.699(1.416  , 5.142) | **0.0025** | 1.716(1.241, 2.373) | **0.0010** | 1.777(1.187  , 2.661) | **0.0052** |
| SAMD4A | 0.932(0.629, 1.380) | 0.7268 |  |  | 1.682(1.137, 2.489) | **0.0092** | 1.770(1.143  , 2.739) | **0.0104** | 1.586(1.205, 2.086) | **0.0009** | 1.604(1.215  , 2.119) | **0.0008** |
| Values expressed as odds ratio (OR) and 95% confidence interval (CI). aOR, adjusted odds ratio; MX2, Myxovirus resistance 2; SAMD4A, Sterile alpha motif domain-containing 4A; HBsAg, hepatitis B surface antigen; HBeAg, Hepatitis B e antigen; ALT, alanine aminotransferase; WBC, white blood cells; PLT: platelet. Bold values are statistically significant P < 0.05. | | | | | | | | | | | | |
