# Supplementary material for: Altered expression of MX2 and SAMD4A in PBMCs predicts early treatment responses in HBeAg-positive chronic hepatitis B patients during Peg-IFN-α therapy
Source: Front Pharmacol. 2026 Jun 22;17:1844257. doi: 10.3389/fphar.2026.1844257 (PMC13333471; doi:10.3389/fphar.2026.1844257)
Supplement: Supplementary file 11 [file Table3.docx]

| **Table S3** Comparison of clinical characteristics between Peg-IFN-α virological response (VR) group and non-virological response (NVR) group | | | | | | | | | | |
| --- | --- | --- | --- | --- | --- | --- | --- | --- | --- | --- |
| characteristics | All | 0w |  | P value | 12w |  | P value | 24w |  | P value |
|  | (n=82) | VR group  (n=53) | NVR group  (n=29) |  | VR group  (n=53) | NVR group  (n=29) |  | VR group  (n=53) | NVR group  (n=29) |  |
| HBsAg (log10 IU/mL) | 3.034(2.068,3.415) | 2.716±  0.9266 | 3.163(2.562,3.456) | 0.3402 | 2.679(1.356,3.152) | 3.030(1.966,3.255) | 0.0648 | 1.751(0.9015,2.985) | 2.953(2.054,3.355) | **0.0034** |
| HBeAg (log10 IU/mL) | 2.571±  0.7623 | 2.645±  0.7825 | 2.435±  0.7171 | 0.2232 | 2.116±0.7778 | 1.878  ±0.6448 | 0.1415 | 2.077(1.033,2.549) | 1.642  ±0.7657 | 0.1950 |
| HBV DNA (log10 IU/mL) | 5.943(5.215,6.639) | 5.428(5.162,5.987) | 6.634(6.187,6.810) | **<0.0001** | 4.215(3.756,4.691) | 5.444  ±0.6542 | **<0.0001** | 3.193(2.420,3.637) | 4.961(4.587,5.651) | **<0.0001** |
| ALT(U/L) | 138.5(121.5,161.0) | 142.0(123.0,162.0) | 132.0(119.0,157.0) | 0.4500 | 72.00(47.00,87.50) | 63.00(44.50,93.00) | 0.5101 | 49.00(30.50,74.50) | 32.00(20.00,61.50) | 0.0662 |
| PLT (×10^9/L) | 156.7  ±65.19 | 156.6  ±67.95 | 156.8±60.98 | 0.9934 | 164.3±64.25 | 161.5±64.64 | 0.8551 | 153.9±67.26 | 172.2  ±68.83 | 0.2502 |
| WBC (×10^9/L) | 4.050(3.160,5.615) | 3.770(3.100,5.540) | 4.699±1.767 | 0.2868 | 4.330(3.240,5.765) | 4.459±1.645 | 0.7743 | 4.270(3.475,5.785) | 4.900(3.430,6.485) | 0.6245 |
| HBsAg, hepatitis B surface antigen; HBeAg, Hepatitis B e antigen; ALT, alanine aminotransferase; WBC: white blood cells; PLT: platelet; VR, virological response; NVR, non-virological response; Bold values are statistically significant P < 0.05. | | | | | | | | | | |
|  | | | | | | | | | | |
