# Supplementary material for: Altered expression of MX2 and SAMD4A in PBMCs predicts early treatment responses in HBeAg-positive chronic hepatitis B patients during Peg-IFN-α therapy
Source: Front Pharmacol. 2026 Jun 22;17:1844257. doi: 10.3389/fphar.2026.1844257 (PMC13333471; doi:10.3389/fphar.2026.1844257)
Supplement: Supplementary file 12 [file Table4.docx]

| **Table S4** Comparison of clinical characteristics between Peg-IFN-α serological response (SR) group and non-serological response (NSR) group | | | | | | | | | | |
| --- | --- | --- | --- | --- | --- | --- | --- | --- | --- | --- |
| characteristics | All | 0w |  | P value | 12w |  | P value | 24w |  | P value |
|  | (n=82) | SR group  (n=32) | NSR group  (n=50) |  | SR group  (n=32) | NSR group  (n=50) |  | SR group  (n=32) | NSR group  (n=50) |  |
| HBsAg (log10 IU/mL) | 3.034(2.068,3.415) | 2.657±  0.8118 | 3.176(2.441,3.432) | 0.1452 | 2.247(1.397,3.174) | 2.941(1.832,3.301) | 0.0950 | 1.679(0.4693,  2.948) | 2.903(1.658,3.265) | **0.0087** |
| HBeAg (log10 IU/mL) | 2.571±  0.7623 | 2.438±  0.8533 | 2.656  ±0.6937 | 0.2316 | 1.735(1.558,1.948) | 2.275  ±0.7668 | **<0.0001** | 1.172  ±0.8586 | 2.133  ±0.7195 | **<0.0001** |
| HBV DNA (log10 IU/mL) | 5.943(5.215,6.639) | 5.789(5.148,6.039) | 6.164(5.401,6.694) | **0.0204** | 4.392  ±1.043 | 4.675(4.199,5.603) | 0.1258 | 3.314(1.699,4.543) | 3.666(3.176,4.768) | **0.0261** |
| ALT(U/L) | 138.5(121.5,161.0) | 127.5(116.0,159.8) | 141.5(125.0,161.3) | 0.1418 | 64.38  ±24.24 | 73.00(50.75,94.00) | 0.1457 | 37.50(29.25,57.75) | 45.00(23.00,91.25) | 0.8261 |
| PLT(×10^9/L) | 156.7  ±65.19 | 156.2  ±62.42 | 157.0  ±67.53 | 0.9560 | 173.2  ±71.31 | 156.9  ±58.73 | 0.2865 | 157.1±65.64 | 162.5  ±69.97 | 0.7261 |
| WBC(×10^9/L) | 4.050(3.160,5.615) | 3.695(3.168,6.098) | 4.391  ±1.612 | 0.7039 | 3.600(2.925,6.175) | 4.595  ±1.354 | 0.8291 | 4.170(3.680,5.885) | 4.655(3.378,6.300) | 0.8151 |
| HBsAg, hepatitis B surface antigen; HBeAg, Hepatitis B e antigen; ALT, alanine aminotransferase; WBC: white blood cells; PLT: platelet; SR, serological response; NSR, non-serological response; Bold values are statistically significant P < 0.05. | | | | | | | | | | |
|  | | | | | | | | | | |
