# Supplementary material for: Altered expression of MX2 and SAMD4A in PBMCs predicts early treatment responses in HBeAg-positive chronic hepatitis B patients during Peg-IFN-α therapy
Source: Front Pharmacol. 2026 Jun 22;17:1844257. doi: 10.3389/fphar.2026.1844257 (PMC13333471; doi:10.3389/fphar.2026.1844257)
Supplement: Supplementary file 13 [file Table11.docx]

| **Table S11** Model performance for SR prediction in the internal validation. | | | | |
| --- | --- | --- | --- | --- |
| Target Gene | Optimism Corrected C-index | Slope | Intercept | Brier Score |
| MX2 | 0.835 | 0.946 | 0.003 | 0.126 |
| SAMD4A | 0.863 | 0.988 | 0.009 | 0.103 |
| SR, serological response; C-index, concordance index. | | | | |
