# Supplementary material for: Altered expression of MX2 and SAMD4A in PBMCs predicts early treatment responses in HBeAg-positive chronic hepatitis B patients during Peg-IFN-α therapy
Source: Front Pharmacol. 2026 Jun 22;17:1844257. doi: 10.3389/fphar.2026.1844257 (PMC13333471; doi:10.3389/fphar.2026.1844257)
Supplement: Supplementary file 14 [file Table6.docx]

| **Table S6** On-treatment variables associated with serological response according to univariate and multivariate analyses | | | | | | | | | | | | |
| --- | --- | --- | --- | --- | --- | --- | --- | --- | --- | --- | --- | --- |
| Variables | Univariate analyses |  | Multivariate analyses |  | Univariate analyses |  | Multivariate analyses |  | Univariate analyses |  | Multivariate analyses |  |
|  | OR(95%CI) | P-value | aOR(95%CI) | P-value | OR(95%CI) | P- value | aOR(95%CI) | P-value | OR(95%CI) | P-value | aOR(95%CI) | P-  value |
|  | **Week0** |  |  |  | **Week12** |  |  |  | **Week24** |  |  |  |
| Gender | 1.201( 0.496, 2.906) | 0.6844 |  |  | 1.201( 0.496, 2.906) | 0.6844 |  |  | 1.201( 0.496, 2.906) | 0.6844 |  |  |
| Age | 1.028(0.984, 1.074) | 0.2100 |  |  | 1.028(0.984, 1.074) | 0.2100 |  |  | 1.028(0.984, 1.074) | 0.2100 |  |  |
| HBsAg | 0.778(0.468, 1.293) | 0.3346 |  |  | 0.727(0.485, 1.090) | 0.1230 |  |  | 0.919(0.441, 1.869) | 0.0556 |  |  |
| HBV DNA | 0.490(0.244, 0.981) | **0.0442** | 0.484(0.235  , 0.995) | **0.0485** | 0.654(0.413, 1.036) | 0.0708 |  |  | 0.629(0.425, 1.929) | 0.2206 |  |  |
| HBeAg | 0.776(0.432, 1.394) | 0.3966 |  |  | 0.268(0.119, 0.601) | **0.0013** | 0.143(0.041  , 0.491) | **0.0020** | 0.212(0.101, 0.446) | **0.0001** | 0.141(0.034  , 0.583) | **0.0068** |
| ALT | 0.993(0.977, 1.009) | 0.4051 |  |  | 0.999(0.990, 1.009) | 0.9610 |  |  | 0.995(0.986, 1.004) | 0.3319 |  |  |
| WBC | 1.096(0.846, 1.421) | 0.4848 |  |  | 0.997(0.760, 1.308) | 0.9882 |  |  | 1.029(0.811, 1.305) | 0.8099 |  |  |
| PLT | 1.002(0.993, 1.007) | 0.9457 |  |  | 1.005(0.997, 1.012) | 0.1747 |  |  | 0.998(0.992, 1.005) | 0.7258 |  |  |

**Continued Table S6** On-treatment variables associated with serological response according to univariate and multivariate analyses

| Variables | Univariate analyses |  | Multivariate analyses |  | Univariate analyses |  | Multivariate analyses |  | Univariate analyses |  | Multivariate analyses |  |
| --- | --- | --- | --- | --- | --- | --- | --- | --- | --- | --- | --- | --- |
|  | OR(95%CI) | P-value | aOR(95%CI) | P-value | OR(95%CI) | P-value | aOR(95%CI) | P-value | OR(95%CI) | P-value | aOR(95%CI) | P-  value |
| MX2 | 0.521(0.226, 1.198) | 0.1253 |  |  | 2.606(1.457  , 4.662) | **0.0012** | 3.654(1.707  , 7.819) | **0.0008** | 1.893(1.421, 2.521) | **0.0010** | 2.255(1.358  , 3.746) | **0.0016** |
| SAMD4A | 1.138(0.776, 1.670) | 0.5063 |  |  | 1.782(1.269, 2.502) | **0.0008** | 2.033(1.322  , 3.127) | **0.0012** | 1.811(1.422, 2.306) | **0.0001** | 1.730(1.278  , 2.341) | **0.0003** |
| Values expressed as odds ratio (OR) and 95% confidence interval (CI). aOR, adjusted odds ratio; MX2, Myxovirus resistance 2; SAMD4A, Sterile alpha motif domain-containing 4A; HBsAg, hepatitis B surface antigen; HBeAg, Hepatitis B e antigen; ALT, alanine aminotransferase; WBC, white blood cells; PLT: platelet. Bold values are statistically significant P < 0.05. | | | | | | | | | | | | |
