# Supplementary material for: Altered expression of MX2 and SAMD4A in PBMCs predicts early treatment responses in HBeAg-positive chronic hepatitis B patients during Peg-IFN-α therapy
Source: Front Pharmacol. 2026 Jun 22;17:1844257. doi: 10.3389/fphar.2026.1844257 (PMC13333471; doi:10.3389/fphar.2026.1844257)
Supplement: Supplementary file 15 [file Table10.docx]

| **Table S10** Model performance for VR prediction in the internal validation. | | | | |
| --- | --- | --- | --- | --- |
| Target Gene | Optimism Corrected C-index | Slope | Intercept | Brier Score |
| MX2 | 0.741 | 0.963 | 0.001 | 0.133 |
| SAMD4A | 0.845 | 0.979 | 0.008 | 0.114 |
| VR, virological response; C-index, concordance index. | | | | |
